# Mitochondrial tRNA^Glu^ 14693A > G Mutation, an “Enhancer” to the Phenotypic Expression of Leber's Hereditary Optic Neuropathy

**DOI:** 10.1002/advs.202401856

**Published:** 2024-09-12

**Authors:** Lihao Jin, Dingyi Gan, Wentao He, Na Wu, Shuchenlu Xiang, Yinsheng Wei, Gilbert Eriani, Yanchun Ji, Min‐Xin Guan, Meng Wang

**Affiliations:** ^1^ Center for Genetic Medicine the Fourth Affiliated Hospital of School of Medicine and International School of Medicine International institutes of Medicine Zhejiang University Yiwu 322000 China; ^2^ Division of Medical Genetics and Genomics The Children's Hospital Zhejiang University School of Medicine and National Clinical Research Center for Child Health Hangzhou 310058 China; ^3^ Institute of Genetics Zhejiang University International School of Medicine Hangzhou 310058 China; ^4^ Architecture et Réactivité de l'ARN UPR9002 Centre National de la Recherche Scientifique Université de Strasbourg Institut de Biologie Moléculaire et Cellulaire 2 allée Konrad Roentgen Strasbourg 67084 France

**Keywords:** apoptosis, leber's hereditary optic neuropathy, mitochondrial tRNA mutation, mitophagy, phenotypic expression

## Abstract

Leber's hereditary optic neuropathy (LHON), a maternally inherited ocular disease, is predominantly caused by mitochondrial DNA (mtDNA) mutations. Mitochondrial tRNA variants are hypothesized to amplify the pathogenic impact of three primary mutations. However, the exact mechanisms remained unclear. In the present study, the synergistic effect of the tRNA^Glu^ 14693A > G and *ND6* 14484T > C mutations in three Chinese families affected by LHON is investigated. The m.14693A > G mutation nearly abolishes the pseudouridinylation at position 55 of tRNA^Glu^, leading to structural abnormalities, decreased stability, aberrant mitochondrial protein synthesis, and increased autophagy. In contrast, the *ND6* 14484T > C mutation predominantly impairs complex I function, resulting in heightened apoptosis and virtually no induction of mitochondrial autophagy compared to control cell lines. The presence of dual mutations in the same cell lines exhibited a coexistence of both upregulated cellular stress responses to mitochondrial damage, indicating a scenario of autophagy and mutation dysregulation within these dual‐mutant cell lines. The data proposes a novel hypothesis that mitochondrial tRNA gene mutations generally lead to increased mitochondrial autophagy, while mutations in genes encoding mitochondrial proteins typically induce apoptosis, shedding light on the intricate interplay between different genetic factors in the manifestation of LHON.

## Graphical abstract

Leber's hereditary optic neuropathy is predominantly caused by mitochondrial DNA mutations. Here, a novel hypothesis is proposed that mitochondrial tRNA gene mutations generally lead to increased mitochondrial autophagy, while the other mutations typically induce apoptosis. The coexistence leads to a heightened cellular stress response and a disruption in the delicate balance between autophagy and apoptosis.

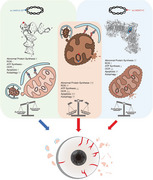

## 1 Introduction

Leber's hereditary optic neuropathy (LHON) is the first disease whose origin has been linked to mitochondrial DNA (mtDNA). It provides a convincing illustration of the complex interaction between genetics and eye health.^[^ ^1^, ^2^, ^3^ ^]^ Characterized by a sudden and often devastating loss of central vision, LHON generally manifests itself in early adulthood, profoundly affecting individuals by progressively diminishing their ability to perceive the details of their environment.^[^ ^3^, ^4^ ^]^ The pathophysiology of the visual impairment is intricately linked to the degeneration of retinal ganglion cells (RGCs), which, for reasons that remain partially elusive, are particularly susceptible to pathological decline.^[^ ^1^, ^5^, ^6^ ^]^

LHON occupies a special place among mitochondrial pathologies, distinguished by its close association with certain mtDNA mutations. In particular, 95% of LHON cases are linked to three primary mutations: m.3460G > A, m.11778G > A, and m.14484T > C.^[^ ^7^, ^8^, ^9^, ^10^, ^11^ ^]^ These genetic alterations are precisely located in the genes encoding ND1, ND4, and ND6 subunits of mitochondrial complex I, which are integral to mitochondrial function.^[^ ^12^ ^]^ The m.14484T > C mutation, which causes a substitution of methionine for valine (M64V), reduces the maximum respiration rate by 10–15%.^[^ ^13^, ^14^ ^]^ Incomplete penetrance was reported in different pedigrees affected by LHON, with some Chinese families showing a low average of 10%,^[^ ^15^ ^]^ while others have rates as high as 60%.^[^ ^16^, ^17^ ^]^ Individuals with the m.14484T > C mutation often experience a later onset of symptoms and a better prognosis compared to other primary LHON mutations.^[^ ^18^ ^]^ Importantly, specific mitochondrial haplogroups, including M9, M10, and N9, and their associated variants – m.3394T > C (*ND1*), m.14502T > C (*ND4*), and m.14693A > G (*TE*) – have been identified as potential amplifiers of optic neuropathy penetrance, which is explained by their combined synergistic effects.^[^ ^19^, ^20^ ^]^ LHON is closely linked to variants in mitochondrial tRNA genes. For example, variants such as tRNA^Met^ 4435A > G and tRNA^Thr^ 15951A > G, which are found associated with the LHON‐associated ND4 m.11778G > A mutation, modulate its phenotypic expression and are considered “LHON modulators”.^[^ ^21^, ^22^, ^23^, ^24^, ^25^ ^]^ In addition, the m.14693A > G variant, located at position 54 of tRNA^Glu^, modifies the tertiary structure of tRNA, potentially modulating the phenotypic manifestation of the LHON‐associated primary mutation m.3460G > A.^[^ ^26^ ^]^ Several other tRNA variants, including in tRNA^Phe^ 593T > C, tRNA^Leu^ 3275C > A, tRNA^Gln^ 4381A > G, tRNA^His^ 12192G > A, and tRNA^Glu^ 14692A > G, have also been associated with LHON, leading to more detailed functional analyses to elucidate their roles.^[^ ^25^, ^27^, ^28^, ^29^ ^]^

Mitochondrial quality control is a vital cellular mechanism that ensures the proper functioning and integrity of mitochondria, the cell's energy‐producing organelles.^[^ ^30^, ^31^, ^32^ ^]^ This process involves several key components, including the surveillance and repair of mtDNA, the regulation of mitochondrial dynamics through fusion and fission, and the selective removal of damaged or dysfunctional mitochondria.^[^ ^30^, ^31^ ^]^ The maintenance of mitochondrial quality is crucial for cellular health, as it prevents the accumulation of defective mitochondria that can lead to impaired energy production and increased oxidative stress.^[^ ^30^, ^33^ ^]^ In the context of LHON, mitochondrial quality control plays a significant role in managing the impact of primary mtDNA mutations. These mutations can impair mitochondrial function, potentially leading to an accumulation of damaged mitochondria.

Mitophagy, a selective form of autophagy that targets these dysfunctional mitochondria for degradation and recycling, becomes a critical process in removing them, thereby preventing excessive production of ROS and protecting cells from oxidative stress.^[^ ^34^, ^35^, ^36^ ^]^ The PINK1‐Parkin pathway is one of the most well‐characterized pathways in mitophagy. Parkin is an E3 ubiquitin ligase that is recruited to mitochondria by Pink1. Once localized to the damaged mitochondria, Parkin mediates the addition of ubiquitin chains to various mitochondrial proteins. This ubiquitination serves as a signal for the recognition and degradation of these mitochondria by the autophagy machinery.^[^ ^36^, ^37^ ^]^ Parkin's activity is crucial for the clearance of dysfunctional mitochondria, thus maintaining mitochondrial quality and preventing the accumulation of mitochondrial damage that can lead to cellular dysfunction. In this process, LC3‐II, a lipidated form of LC3 that associates with autophagosomal membranes, plays a critical role. Following the completion of the autophagosome, LC3‐II is cleaved by the cysteine protease Atg4B and recycled, allowing for the continuous formation of new autophagosomes. Additionally, SQSTM1/p62, a well‐known autophagic substrate, interacts with LC3 to ensure the selective delivery of ubiquitinated proteins into the autophagosome.^[^ ^36^, ^37^, ^38^ ^]^ This interaction facilitates the targeted degradation of damaged mitochondria, further enhancing the specificity and efficiency of the mitophagy process.

Apoptosis, or programmed cell death, is a complex process influenced by various factors, including cellular stress, DNA damage, and mitochondrial dysfunction.^[^ ^39^, ^40^ ^]^ Central to the regulation of apoptosis are several key proteins, each playing a distinct role in the apoptotic cascade. Proteins such as Bcl‐XL and BAX are part of the Bcl‐2 family, which governs mitochondrial outer membrane permeabilization (MOMP), a critical event in apoptosis. Bcl‐XL acts as an anti‐apoptotic protein, inhibiting the release of pro‐apoptotic factors, while BAX promotes apoptosis by facilitating MOMP.^[^ ^41^, ^42^ ^]^ BAD, another member of the Bcl‐2 family, can promote apoptosis by antagonizing the function of anti‐apoptotic proteins. Upon activation, it contributes to the release of cytochrome c (CytC) from mitochondria into the cytosol. CytC then binds to Apaf‐1, leading to the formation of the apoptosome and the activation of initiator caspases like Caspase9. This activation triggers a cascade involving caspase executors such as Caspase3, leading to the systematic dismantling of cell components.^[^ ^40^, ^43^ ^]^ In the context of LHON, mitochondrial dysfunction plays a pivotal role in triggering apoptosis. The primary mtDNA mutations associated with LHON impair mitochondrial function, leading to increased oxidative stress, which could initiate apoptotic pathways, particularly involving proteins like BAX, BAD, and CytC, contributing to the loss of RGCs.^[^ ^34^, ^39^ ^]^

In present study, we identified three pedigrees carrying both the m.14484T > C and m.14693A > G mutations. Compared to typical m.14484T > C patients, these individuals exhibited earlier onset and more severe symptoms. We extracted peripheral blood from patient ZJL847‐III‐2 to establish a lymphoblastoid cell line and subsequently created cybrid cell lines. Concurrently, we constructed control cell lines (C101) with a similar haplotype but without any mitochondrial mutations, as well as cell lines carrying only the m.14693A > G mutation in tRNA^Glu^ (HZL017‐III‐3) or only the m.14484T > C mutation in ND6 (ZJL855‐III‐3). Our research revealed that the m.14693A > G and m.14484T > C mutations independently manage mitochondrial stress through mitochondrial autophagy and apoptosis, respectively. However, in the double‐mutated cell lines, both pathways were significantly upregulated. This finding elucidates why patients with dual mutations exhibit higher penetrance and severity of LHON. It also suggests the hypothesis that mutations in mitochondrial tRNA genes and those encoding mitochondrial proteins may cause different mitochondrial stresses, leading to varied cellular stress responses.

## 2 Results

### 2.1 Mitochondrial tRNA Evaluation

#### 2.1.1 Clinical Genetic Evaluation of Three Chinese Families Carrying both m.14693A > G and m.14484T > C Mutation

**Figure 1:**
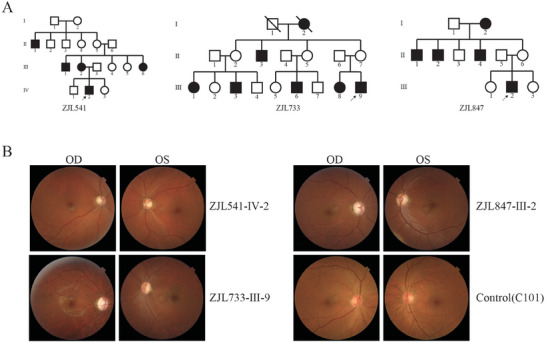
Clinical and genetic characterization of eight Chinese families. A) LHON in three Han Chinese families. Vision‐impaired individuals are indicated by black symbols. B) Fundus photographs (Cannon CR6‐45NM) of Chinese patients, carriers, and control subjects.

In a comprehensive survey of 1793 Chinese LHON probands, three Han Chinese families were identified, each carrying both tRNA^Glu^ 14693A > G and *ND6* m.14484T > C mutations.^[^ ^8^, ^21^, ^44^ ^]^ Among 37 matrilineal relatives in these families, 17 exhibited varying degrees of penetrance and expression of optic neuropathy, with the severity of vision loss ranging from profound to severe (Table S1, Supporting Information; **Figure** **1**A,B). The age of onset of optic neuropathy in three matrilineal relatives with both m.14693A > G and m.14484T > C mutation was 5, 7, and 11 years respectively, with an average of 8 years. The penetrance rate of optic neuropathy in these pedigrees was variable, ranging from 35.7% to 55.6%, with an average of 47.1%. Furthermore, we identified 22 patients with m.14693A > G mutation in 811 LHON patients, including these three probands. The penetrance rate of optic neuropathy in these pedigrees m.14693A > G mutation only without m.14484T > C mutation ranges from 4.3% to 20.0%, with an average of 7.8%. The age of onset of optic neuropathy ranges from 18 to 55, with an average of 38.^[^ ^8^ ^]^ In a previous study, we examined LHON families carrying the m.14484T > C mutation and found that the penetrance rate of optic neuropathy in these pedigrees carrying m.14484T > C mutation only without m.14693A > G mutation ranged from 4.8% to 60%, with an average of 17.7%. The age of onset of optic neuropathy ranged from 3 to 35, with an average of 15,^[^ ^19^ ^]^ suggesting that the m.14693A > G mutation is likely to increase LHON penetrance. In addition, a previous study showed the function of the m.14693A > G mutation as an enhancer, impacting the phenotype of ND1 m.3460G > A mutation, with penetrance reaching 43%.^[^ ^45^ ^]^

Regarding the matrilineal relatives identified here, they had no other clinical abnormalities, such as cardiac failure, muscular diseases, or neurological disorders. Further genetic analysis revealed that the tRNA^Glu^ 14693A > G mutation was homoplasmic in all matrilineal relatives but was absent in other family members, highlighting its specific association with the observed optic neuropathy (additional data not presented).

#### 2.1.2 Steady State Levels of tRNA^Glu^

To assess whether the m.14693A > G mutation affected tRNA^Glu^ metabolism, we subjected mitochondrial RNAs from mutant and control cybrid cell lines to Northern blots and hybridized them with DIG‐labeled oligodeoxynucleotide probes for tRNA^Glu^, tRNA^Ser(AGY)^ and tRNA^Lys^. As shown in Figure S1A,B (Supporting Information), the steady‐state levels of tRNAs in the mutant cells, across all variants, exhibited no significant differences when compared to the control cells.

#### 2.1.3 Defect of Ψ55 Modification and Perturbed Conformation of tRNA^Glu^

**Figure 2:**
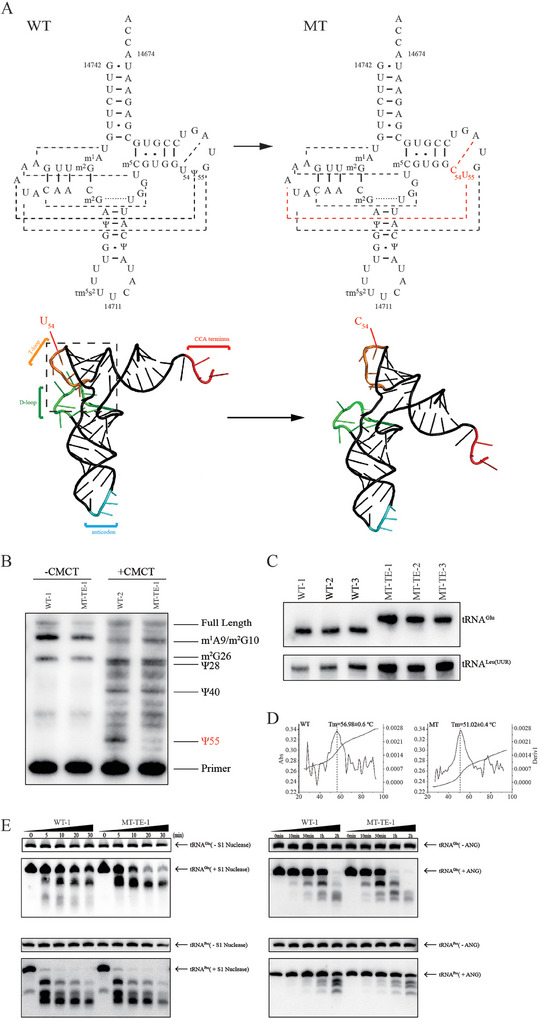
Alteration in the structure and conformation of mitochondrial tRNA^Glu^. A) Cloverleaf structure and Schematic of the tertiary structure of wild type and mutant human mitochondrial tRNA^Glu^. Red dashed lines represent the anticipated disturbances in tertiary base pairings. B) Mitochondrial RNA from control (C101.2) and mutant (HZL017‐III‐3.3) cells were incubated with CMCT for CMC modification of Ψ residues. Reverse transcription was carried out to identify the stops caused by CMC‐pseudouridine. C) Assessment of conformation changes by PAGE analysis under native conditions. D) Melting profiles of WT and MT tRNA^Glu^ transcripts measured at 260 nm with a heating rate of 1° min^−1^ from 25 to 95°. First derivative (dA/dT) against temperature curves was shown to highlight the Tm value transitions. E) S1 and angiogenin digestion pattern of tRNA^Glu^ and tRNA^Pro^ purified from the control cell line (C101.2) and mutant cell line (HZL017‐III‐3.3). Cleavage products of tRNAs were resolved in 10% desaturating PAGE gels with 8 M urea, electroblotted, and hybridized with specific 3′ end DIG‐labeled oligonucleotide probes.

Given the critical role of the reverse Hoogstein base pair U54‐A58 for U55 pseudouridinylation of tRNAs,^[^ ^46^ ^]^ we might expect the m.14693A > G mutation leading to U54C substitution to alter adjacent U55 modification. Indeed, the mutation should destabilize the A18‐U55 base pairing between the T‐loop and D‐loop of tRNA^Glu^ and reduce the Ψ55 modification in tRNA^Glu^ thereby altering the tertiary structure and function of tRNA^Glu^ (**Figure** **2**A).

To study the impact of the m.14693A > G mutation on tRNA^Glu^ pseudouridinylation, we treated the total mitochondrial tRNA extract by CMCT (1‐cyclohexyl‐(2‐morpholinoethyl)carbodiimide metho‐p‐toluene sulfonate) followed by reverse transcription with a specific DIG‐labeled oligonucleotide specific of tRNA^Glu^. The carbodiimide (CMC) moiety of CMCT modifies the N1 of guanosine, the N3 of uridine, and the N1 and N3 of pseudouridine. The subsequent mild alkaline treatment removes the adducts from uridine and guanosine, but not from N3‐CMC‐pseudouridine. The bulky CMC modification acts as a barrier for reverse transcriptase, creating a distinct stop on the sequence gel, one nucleotide in 3′ of pseudouridine. The results, shown in Figure 2B, reveal a significant reduction in Ψ55 modification in tRNA^Glu^ from the mutant cell line (m.14693A > G, MT‐TE), in stark contrast to the control cell lines (C101, WT), where the Ψ55 modification is well detected.

#### 2.1.4 Alterations in Stability and Conformation of tRNA^Glu^

To test whether the m.14693A > G mutation affects the conformation of tRNA^Glu^, total mitochondrial RNA extracts were electrophoresed through an 8% polyacrylamide gel (native condition) in 25 mM Tris‐Glycine buffer and then electroblotted onto a positively charged nylon membrane for hybridization analysis with oligodeoxynucleotide probes for tRNA^Glu^ and tRNA^Leu (UUR)^, respectively. Migration profiles showed that tRNA^Glu^ from three mutant cybrid cell lines carrying the m.14693A > G mutation migrate much slower than those of control cybrid cell lines lacking this mutation (Figure 2C). These data indicate that the m.14693A > G mutation alters the conformation of tRNA^Glu^.

The folding stability of wild‐type and mutant tRNA^Glu^ transcripts was assessed by measuring melting temperatures (Tm) and calculating absorbance derivatives against a temperature curve. Tm values for wild‐type and mutant transcripts were 56.98 ± 0.6°C and 51.02 ± 0.4°C, respectively (Figure 2D). This shows that the m.14693A > G mutation affects the stability of tRNA^Glu^. We validated this hypothesis using Angiogenin (ANG) and S1 nuclease cleavage assays. Cleaved products from control and mutant cell lines were analyzed by Northern blot using tRNA^Glu^‐specific probe hybridizing to the 3′ half of the tRNA. The gels shown in Figure 2E demonstrate that, at equivalent nuclease dilution, tRNA^Glu^ from mutant cell lines was more cleaved by S1 nuclease and ANG than those from control cell lines. Conversely, there was no significant difference in the cleavage profiles of a control tRNA^Pro^ in the same cell lines. These data validated that the m.14693 > G changed the conformation of mitochondrial tRNA^Glu^.

### 2.2 Assessment of Mitochondrial Function

**Figure 3:**
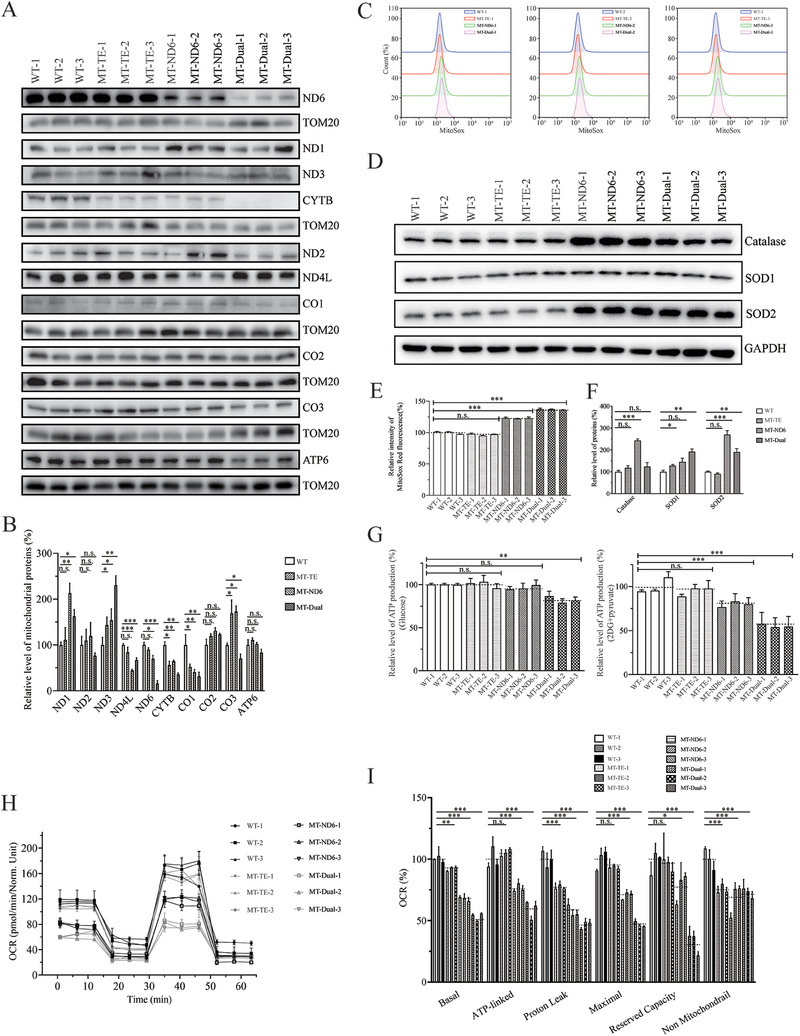
Assessment of mitochondrial function. A) Western blot analysis of mitochondrial proteins. 20 µg of total cellular proteins from various cell lines were electrophoresed through a denaturing polyacrylamide gel, electroblotted, and immuno‐detected with ten respiratory complex subunits specific antibodies in mutant and control cells with TOM20 as a loading control. B) Quantification of OXPHOS subunits levels in control and mutant cell lines. C) Measurement of mitochondrial ROS. The rates of ROS generation by mitochondria in living cells from mutant and control cell lines were analyzed by a Novocyte flow cytometer (ACEA Biosciences) using the mitochondrial superoxide indicator MitoSOX‐Red (5 mm). A, flow cytometry histogram showing MitoSOX‐Red fluorescence of various cell lines. D) Western blot analysis of three antioxidative enzymes. 20µg of total proteins from various cell lines was electrophoresed, electroblotted, and hybridized with catalase, SOD1, and SOD2 antibodies and with GAPDH as a loading control. E) Relative ratios of MitoSOX‐Red fluorescence intensity. F) quantification of SOD2, SOD1, and Catalase. Average relative values of SOD2, SOD1, and Catalase were normalized to the average values of GAPDH in various cell lines. G) Measurement of cellular and mitochondrial ATP levels using a bioluminescence assay. Cells were incubated with 10 mm glucose or 5 mm 2‐deoxy‐d‐glucose plus 5 mm pyruvate to determine ATP generation under mitochondrial ATP synthesis. Average rates of ATP level per cell line and are shown. H) An analysis of O2 consumption rate (OCR) in the various cell lines using different inhibitors. The OCRs were first measured on 1 × 10^4^ cells of each cell line under basal condition and then sequentially added oligomycin (1.5 µM), carbonyl cyanide p‐(trifluoromethoxy)phenylhydrazone (FCCP) (0.5 µM), rotenone (1 µM) and antimycin A (1 µM) at indicated times to determine different parameters of mitochondrial functions. I) Graphs presented the ATP‐linked OCR, proton leak OCR, maximal OCR, reserve capacity OCR, and non‐mitochondrial OCR in mutant and control cell lines. Non‐mitochondrial OCR was determined as the OCR after rotenone/antimycin A treatment. Basal OCR was determined as OCR before oligomycin minus OCR after rotenone/antimycin A. ATP‐linked OCR was determined as OCR before oligomycin minus OCR after oligomycin. Proton leak OCR was determined as basal OCR minus ATP‐linked OCR. Maximal OCR was determined as the OCR after FCCP minus non‐mitochondrial OCR. Reserve capacity OCR was defined as the difference between maximal OCR after FCCP minus basal OCR. The average values of three determinations for each cell line are shown. Data are represented as mean ± SEM. Statistical significance is indicated as follows: n.s., no significance, *p < 0.05, ***p < 0.01, ***p < 0.001. Asterisks represent the level of significance in comparison to the control group.

To explore the impacts of the m.14693A > G and m.14484T > C mutations on the biogenesis of oxidative phosphorylation (OXPHOS) machinery subunits, we conducted Western blot analyses focusing on 10 mitochondrial DNA‐encoded proteins in the mutant cell lines carrying only the tRNA^Glu^ 14693A > G mutation (MT‐TE), or only the *ND6* m.14484T > C mutation (MT‐ND6), or both m.14693A > G and m.14484T > C mutations (MT‐DUAL), as well as control cell line (C101, WT) lacking these mutations. The analysis, described in **Figure** **3**A,B, revealed a marked variation in the expression levels of eight of these proteins, except CO2 and ATP6 which remained unchanged. Overall levels of mitochondrial translation products showed different patterns, while dual mutation cell lines, harboring both m.14693A > G and m.14484T > C mutations, showed a more pronounced reduction in mitochondrial translation products. It appears that 4 proteins (ND4L, ND6, CYTB, CO1) are systematically strongly reduced in the 3 cell lines, while 3 proteins (ND1, ND3, CO3) are strongly upregulated in the mutated cell lines, particularly in the presence of the *ND6* mutation. It is also worth noting that the double mutant cell line often shows a greater decrease in mitochondrial proteins than the single mutant cell lines. This indicates a combined effect on mitochondrial translation in the presence of both mutations, suggesting a synergistic effect of m.14693A > G and m.14484T > C on mitochondrial protein synthesis, which could potentially lead to functional deficits in the mitochondrial respiratory chain.

#### 2.2.1 The Increase of ROS Production

Mitochondrial reactive oxygen species (ROS) play critical roles in physiological in varied cellular processes.^[^ ^47^, ^48^, ^49^ ^]^ ROS production was assessed in mutant and control cybrid cell lines via flow cytometry, comparing baseline staining intensity for each cell line with that upon oxidative stress to obtain a ratio corresponding to ROS generation.^[^ ^50^, ^51^, ^52^ ^]^ Geometric mean intensity was recorded to measure the rate of mitochondrial ROS of each sample. The relative levels of geometric mean intensity in each cell line were calculated to delineate the levels of mitochondrial ROS in mutant and control cells. As shown in Figure 3C,E, the levels of ROS generation in MT‐TE, MT‐ND6, and MT‐DUAL cybrids were 96.7%, 122.5%, and 136.8% of the mean values measured in the control cell lines. Furthermore, we examined the levels of catalase and superoxide dismutase proteins (SOD2 and SOD1) by western blot analysis.^[^ ^49^, ^53^ ^]^ As shown in Figure 3D,F, a marked increase of the levels of these proteins was observed in the mutant cybrids. In particular, the levels of catalase, SOD1, and SOD2 in MT‐TE cybrids were 118.6%, 242.8%, and 123.6%, while those in MT‐ND6 cybrids were 242.8%, 145.6%, and 270.0%, and 123.6%, 192.1%, and 190.3% in MT‐DUAL cybrids, respectively, relative to the mean values measured in the control cell lines.

#### 2.2.2 Reduced Level in Mitochondrial ATP Production

To examine the capacity of OXPHOS, we measured the levels of cellular and mitochondrial ATP production using a luciferin/luciferase assay.^[^ ^54^ ^]^ Cell populations were incubated in the media in the presence of glucose and 2‐deoxy‐d‐glucose with pyruvate to inhibit glycolysis. As shown in Figure 3G, the levels of mitochondrial ATP production (pyruvate and 2‐deoxy‐d‐glucose) in the MT‐TE, MT‐ND6, and MT‐DUAL cybrids were 94.7%, 79.8%, and 55.3%, respectively, of the mean values for control cybrids, while levels of cellular ATP production (glucose) in mutant cybrids were comparable to those measured in control cybrids.

#### 2.2.3 Respiration Deficiency

To further examine whether the mutations affected cellular bioenergetics, we examined the oxygen consumption rates (OCR) of various mutant and control cybrid cell lines with a Seahorse Bioscience XF‐96 extracellular flux analyzer (Seahorse Bioscience).^[^ ^54^, ^55^ ^]^ As shown in Figure 3H,I, the basal OCR in the MT‐TE, MT‐ND6 and MT‐DUAL cybrids were 92.3%, 67.7%, and 53.4%, respectively, relative to the mean value measured in the control cybrids. To determine which of the of the respiratory chain enzyme complexes was perturbed in the mutant cell lines, the OCR was measured after the sequential addition of oligomycin (inhibitor of the ATP synthase), carbonyl cyanide p‐(trifluoromethoxy) phenylhydrazone (FCCP; to uncouple the mitochondrial inner membrane and allow for maximum electron flux through the electron transport chain), rotenone (to inhibit complex I), and antimycin (to inhibit complex III). The difference between basal OCR and drug‐resistant OCR yielded an amount of ATP‐linked OCR, proton leak OCR, maximal OCR, reserve capacity, and nonmitochondrial OCR. As shown in Figure 3H,I, the ATP‐linked OCR, proton leak OCR, maximal OCR, reserve capacity, and nonmitochondrial OCR in MT‐DUAL cybrids were 59.1%, 46.8%, 47.0%, 32.3%, and 72.8%, respectively, those in MT‐TE cybrids were 105.3%, 77.2%, 93.3%, 95.6%, and 75.4%, respectively, and those in MT‐ND6 cybrids were 76.4%, 57.5%, 70.7%, 77.5%, and 68.1%, respectively, relative to the mean values measured in the control cybrids.

### 2.3 Investigation of Mitochondrial Quality Control

#### 2.3.1 Promoting Apoptosis

**Figure 4:**
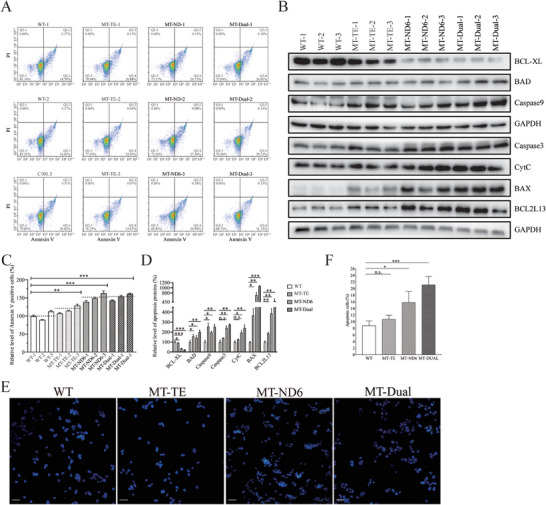
Investigation of apoptosis. A) Annexin V/PI apoptosis assay by flow cytometry. Cells were harvested and stained with Annexin V and 1 µL of PI. Flow cytometric plots show cells in the live, early apoptosis, and late apoptosis stages. Apoptosis rate increased in mutant cells compared with control cells. (B) Western blot analysis of apoptosis related proteins. 20 µg of total proteins from various cell lines were electrophoresed, electroblotted, and hybridized with Bcl‐XL, BAD, Caspase 9, Caspase 3, CytC, BAX, BCL2L13 antibodies and with GAPDH as a loading control. C) Relative Annexin V‐positive cells from various cell lines. Three independent determinations were done in each cell line. D) Quantification of seven proteins associated with apoptosis. Three independent determinations were done in each cell line. E) Representative images of TUNEL assay in control and mutant cells. F) Quantification of apoptotic cells in control and mutant cell lines. Graph details and symbols as in Figure 3.

Deficient activities of oxidative phosphorylation have been linked to protection against certain apoptotic stimuli.^[^ ^52^, ^56^ ^]^ To assess whether the m.14693A > G and m.14484T > C mutations affected the apoptotic processes, we examined the apoptotic state of our cell lines using Annexin V/PI‐based flow cytometry and Western blot analyses. As shown in **Figure** **4**A,C, the average ratio of Annexin V‐positive cells in the MT‐TE, MT‐ND6, and MT‐DUAL cybrids were 116.6%, 150.3%, and 152.5% of the mean values measured in control cell lines. Furthermore, we examined the levels of three apoptosis‐related proteins (BCL2‐like 1 [Bcl‐XL], BCL2‐associated agonist of cell death [BAD], Caspase 9, Caspase 3, BCL2‐associated X protein [BAX] and BCL2‐like 13 [BCL2L13]) by Western blot analysis. As shown in Figure 4B,D, the marked increase in levels of these proteins was observed in the mutant cybrids excluding Bcl‐XL. In particular, the levels of Bcl‐XL, BAD, Caspase 9, Caspase 3, CytC, BAX and BCL2L13 in MT‐DUAL cybrids were 24.2%, 200.5%, 253.0%, 275.3%, 241.1%, 1063.9%, and 484.9%, those in MT‐TE cybrids were 190.9%, 155.6%, 255.7%, 139.2%, 127.1%, 366.3%, and 185.6%, and those in MT‐ND6 cybrids were 35.0%, 143.6%, 196.6%, 241.9, 194.5%, 718.1%, and 388.3% relative to the mean values measured in the control cybrids, respectively. Afterwards, the TUNEL assay was carried out to assess the apoptosis levels in mutant and control cell lines. As shown in Figure 4E,F, the ratio of apoptotic cells in the MT‐TE cybrids was comparable with that in WT. Increased ratios of apoptotic cells were found in MT‐ND6 and MT‐DUAL cybrids compared with WT. In particular, ratios of apoptotic cells in WT, MT‐TE, MT‐ND6, and MT‐DUAL cybrids were 8.8%, 10.8%, 15.8%, and 21.1%, respectively.

#### 2.3.2 Impairment of Mitophagy

**Figure 5:**
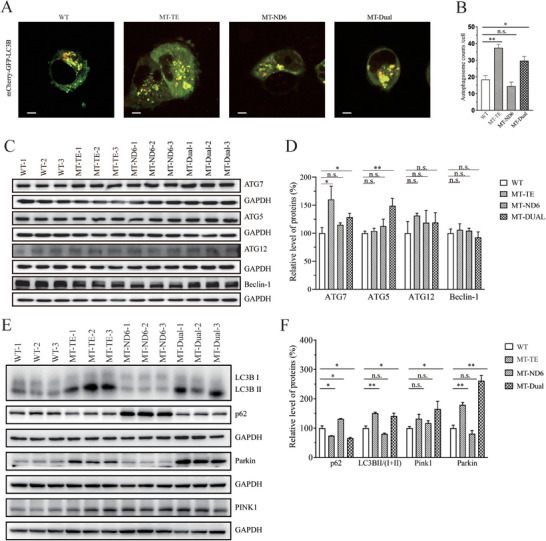
Evaluation of autophagy. A) Representative images of mCherry‐GFP‐LC3B transfection in various cell lines. B) Autophagic flux was quantitatively assessed by counting the number of GFP and mCherry puncta per cell in live images. A minimum of 50 cells per treatment group was analyzed. C) Western blot analysis for ATG5, ATG7, ATG12 and Beclin‐1. 20 µg of total cellular proteins from various cell lines was electrophoresed, electroblotted, and hybridized with ATG5, ATG7, ATG12, and Beclin‐1 antibodies, with GAPDH as a loading control. D) Quantification of four autophagy related proteins, ATG5, ATG7, ATG12, and Beclin‐1 in mutant and control cell lines. E) Western blot analysis for mitophagic response proteins LC3‐I/(I+II), p62, Parkin, and PINK1. 20 µg of total cellular proteins from various cell lines was electrophoresed, electroblotted, and hybridized with LC3, p62, Parkin, and PINK1 antibodies, with GAPDH as a loading control. F) Quantification of four autophagy related proteins, LC3, p62, Parkin, and PINK1 in mutant and control cell lines. Graph details and symbols as in Figure 3.

The alterations in OXPHOS and mitochondrial membrane potential affect the mitophagic elimination of damaged mitochondria.^[^ ^56^, ^57^, ^58^ ^]^ Utilizing the mCherry‐GFP‐LC3B plasmid, we quantitatively assessed the impact of the m.14693A > G and m.14484T > C mutations on autophagic activity in our cell lines. Cells with the sole tRNA^Glu^ 14693A > G mutation had a significantly higher number of autophagosomes than the other groups (**Figure** **5**A,B). The cell lines with the double mutation displayed a moderately high number of autophagosomes. In contrast, the cell lines carrying only the *ND6* m.14484T > C mutation had the lowest number of autophagosomes. These findings suggest a differential modulation of autophagic processes by the m.14693A > G and m.14484T > C mutations, with the former potentially inducing a more robust autophagic response. To further assess the effect of these mutations on mitophagy, we performed a Western blot analysis of the autophagy related proteins ATG5, ATG7, ATG12, Beclin‐1, LC3, SQSTM1/p62, Parkin, and PINK1. The results, illustrated in Figure 5C–F, revealed distinct patterns in the mutant cybrids compared to the controls. Overall, the levels of ATG5, ATG7, ATG12, and Beclin‐1 in MT‐ND6 cybrids were comparable with WT. Increase levels of ATG7 were found in MT‐TE and MT‐DUAL cybrids. In particular, the levels of ATG7 in MT‐TE, MT‐ND6, and MT‐DUAL cybrids were 160.2%, 115.0%, and 128.6%, respectively, relative to the mean values measured in the control cybrids. Levels of ATG5 in these mutant lines were 104.0%, 112.8%, and 149.0% of control values, respectively. Levels of ATG12 in these mutant lines were 131.5%, 118.9%, and 119.0% of control values, respectively. Levels of Beclin‐1 in these mutant lines were 106.1%, 104.5%, and 92.5% of control values, respectively. The LC3‐II/I+II ratios in MT‐TE, MT‐ND6, and MT‐DUAL cybrids were 151.1%, 80.6%, and 141.0%, respectively, of the control values. Levels of p62 in these mutant lines were 74.1%, 131.6%, and 65.9% of control values, respectively. PINK1 levels were 131.9%, 117.0%, and 165.2%, and Parkin levels were 179.1%, 81.2%, and 261.3% of the control values for the respective mutations.

## 3 Discussion

Besides three primary mutations located in the genes encoding ND1, ND4, and ND6 subunits of mitochondrial complex I, mitochondrial tRNA variants have been identified as potential amplifiers of optic neuropathy penetrance and expressivity. However, the exact mechanisms by which the tRNA gene variant exacerbates the effects of the primary mutations remained unclear. In present study, the mitochondrial tRNA^Glu^ 14693A > G mutation was found to disrupt pseudouridinylation at position 55. This modification, universally conserved across life forms, plays a pivotal role in stabilizing the L‐shaped structure of tRNA by forming a tertiary base pair with the conserved 18A/G in the D‐loop. The loss of this modification, as evidenced by our study, leads to structural and functional instability in mutant mitochondrial tRNA^Glu^. This instability is further highlighted by changes in electrophoretic mobility and increased sensitivity to S1 nuclease and ANG‐mediated cleavage. Moreover, the m.14693A > G mutation disrupts the A58‐U54 base pairing, causing more pronounced structural alterations in tRNA^Glu^ than the m.14692A > G mutation associated with diabetes and deafness (Figure S2, Supporting Information).^[^ ^59^ ^]^ These structural alterations likely hinder mitochondrial protein synthesis, which was supported by Western blot analysis. Interestingly, despite these disruptions, cells harboring the m.14693A > G mutation exhibit an effective compensatory response through mitophagy, due to the overall stress it imposes on mitochondrial synthesis. This mechanism is so proficient that it maintains mitochondrial ROS levels comparable to those in control cell lines, underscoring the pivotal role of mitophagy in preserving mitochondrial homeostasis and mitigating oxidative stress under mutational stress. The observed upregulation of PINK1 and Parkin levels specifically indicates the activation of mitophagy, targeting the selective degradation of damaged mitochondria.^[^ ^60^, ^61^ ^]^ Furthermore, the decrease in p62 levels suggests an efficient autophagic degradation process. p62 acts as a crucial adaptor, linking LC3 and ubiquitinated substrates for autophagosomes degradation. Its reduction implies successful delivery and breakdown of these substrates within autolysosomes.^[^ ^62^, ^63^, ^64^ ^]^ Concurrently, the increase in LC3‐II levels is indicative of enhanced autophagosome formation, a hallmark of autophagy activation. As an essential autophagy effector enzyme that in concert with ATG5 and ATG12 proteins, ATG7 plays a central role in autophagosome biogenesis. Increased level of ATG7 implicated that the m.14693A > G mutation promoted the formation and maturation of autophagosome. Collectively, these protein level changes reflect a robust cellular adaptation to mitochondrial stress.^[^ ^62^, ^65^, ^66^, ^67^ ^]^ The cells appear to be actively compensating for mitochondrial dysfunction by augmenting the clearance of impaired mitochondria via mitophagy. This response not only highlights the cellular resilience in the face of mitochondrial perturbations but also underscores the intricate balance between mitochondrial dysfunction and the autophagic response. Upon the activation of mitophagy, a notable observation in our study is the ability of cells exclusively harboring the m.14693A > G mutation to maintain sufficient mitochondrial ATP production. This resilience plays a crucial role in mitigating the cellular stress induced by the m.14693A > G mutation alone, thereby preventing the onset of functional abnormalities within these cells. Importantly, this finding is particularly relevant in the context of RGCs, which are known to have some of the highest ATP demands within the retina. The maintenance of robust mitochondrial function is essential for the optimal performance of RGCs, underscoring the significance of efficient mitochondrial ATP production in these cells.^[^ ^68^, ^69^, ^70^ ^]^

Meanwhile, cell lines with the m.14484T > C mutation, showed a functional impairment of Complex I, leading to a decrease in ATP production and an increase in the generation of ROS, thereby resulting in the release of cytochrome c and subsequent cellular apoptosis. This direct stress on mitochondrial proteins and complexes might induce cells to regulate through apoptosis, potentially causing an increase in hydrogen peroxide levels that significantly elevates Catalase levels compared to other cell lines. Our findings are consistent with previous research,^[^ ^71^, ^72^ ^]^ further corroborating the critical impact of this mutation on mitochondrial function and its subsequent effects on cellular health.

Our results demonstrate an amplified effect on mitochondrial function when tRNA^Glu^ 14693A > G and the LHON *ND6* primary mutation m.14484T > C coexist. This coexistence leads to a heightened cellular stress response, characterized by increased ROS production, reduced mitochondrial ATP production, and respiratory deficiency. A particularly noteworthy observation in our study is the markedly high levels of both autophagy and apoptosis observed in the cell lines harboring both the m.14693A > G and m.14484T > C mutations. This phenomenon suggests a disruption in the delicate balance between these two crucial cellular processes. In typical cellular homeostasis, autophagy and apoptosis function as complementary mechanisms: autophagy primarily serves as a survival pathway, mitigating stress by degrading and recycling cellular components, while apoptosis eliminates cells that are damaged beyond repair.^[^ ^73^, ^74^ ^]^ However, in the dual mutation cell lines, we observed an amplification of both pathways, indicating a cellular environment overwhelmed by stress and dysfunction. Mitophagy, initiated by the ATP‐dependent tagging of damaged mitochondria by PINK1 and Parkin, leads to the formation of an autophagosome and its subsequent fusion with a lysosome to form an autolysosome. Each of these steps, from membrane assembly and elongation to the fusion process and the eventual degradation and recycling of mitochondrial components, consumes a great deal of energy. This continuous high‐energy demand, especially in a scenario where mitochondrial function is already compromised, could exacerbate cellular stress. The sustained activation of both autophagy and apoptosis, therefore, not only reflects a cellular attempt to maintain homeostasis but also highlights the potential for an energy crisis, further impairing cell viability. In the context of LHON patients with dual mutations, this energy crisis becomes a critical molecular mechanism contributing to the early onset, increased penetrance, and more severe phenotype of the disease. The dual mutations exacerbate mitochondrial dysfunction, leading to an increased burden on cellular energy systems. This heightened stress and energy demand may accelerate the degeneration of retinal ganglion cells, thereby intensifying the clinical manifestations of LHON.

The study underscores the complex interplay between different mitochondrial mutations in LHON. While the primary LHON mutations predominantly impair complex I function, leading to apoptosis, the tRNA^Glu^ 14693A > G mutation primarily induces mitochondrial autophagy. This differential modulation of cellular pathways by various mitochondrial mutations provides a deeper understanding of the molecular mechanisms underlying LHON. The balance between these two processes is crucial; while autophagy serves as a cellular protective mechanism by degrading damaged mitochondria and preventing excessive apoptosis, uncontrolled autophagy can itself lead to cell death.^[^ ^73^, ^75^, ^76^, ^77^ ^]^ Conversely, while apoptosis eliminates cells that are irreversibly damaged, excessive apoptosis can result in unnecessary cell loss.^[^ ^73^ ^]^ Understanding how different LHON mutations tip this balance provides valuable insights into the disease's pathogenesis and opens avenues for targeted therapeutic strategies. Future research and potential treatments for LHON may benefit from focusing on how to optimize this balance. Therapeutic approaches that can modulate either autophagy or apoptosis, or both, in a way that compensates for the specific mitochondrial dysfunction caused by different LHON mutations, could be particularly effective. This could involve strategies to enhance mitochondrial function, reduce oxidative stress, or directly target the molecular pathways involved in autophagy and apoptosis. Such approaches hold promise not only for treating LHON but also for providing broader insights into mitochondrial diseases and the intricate mechanisms of cellular maintenance.

In conclusion, this study provides novel insights into the multifaceted role of the mitochondrial tRNA^Glu^ 14693A > G mutation in exacerbating the phenotypic expression of LHON. Our findings illustrate that this mutation significantly impacts mitochondrial function, highlighting its potential as a critical “enhancer” in the pathogenesis of LHON. Furthermore, our research reveals the molecular mechanisms underlying the early onset, increased penetrance, and more severe phenotype of LHON in patients harboring dual mutations. These findings offer a deeper understanding of the potential causes of LHON pathogenesis related to mitochondrial tRNA mutations, shedding light on the intricate interplay between different genetic factors in the manifestation of this condition.

## 4 Experimental Section

### Families and Subjects

In this investigation, three distinct Han Chinese families were engaged, each carrying both m.14693A > G and m.114484T > C mutation, sourced from various ophthalmology clinics throughout China. Extensive medical histories and physical assessments were conducted for all participants, focusing on identifying any history of visual impairment or other health anomalies. We adhered to previously established protocols for ophthalmic examinations of all family members.^[^ ^44^, ^78^ ^]^ Visual acuity was categorized into five levels: normal (above 0.3), mild impairment (between 0.3 and 0.1), moderate impairment (from less than 0.1 to 0.05), severe impairment (from less than 0.05 to 0.02), and profound impairment (below 0.02). Our study strictly followed the guidelines of the Declaration of Helsinki. Ethical clearance was obtained from the Zhejiang University School of Medicine's ethics committee (2021‐IRBAL‐049), and all participants provided informed consent and contributed blood samples for clinical evaluation.

### Mutational Analysis of the Mitochondrial Genome

Genomic DNA was isolated from the whole blood of participants (Puregene DNA Isolation Kit; Gentra Systems, Minneapolis, MN). The presence of m.14693A > G and m.14484T > C mutations were examined as detailed elsewhere.^[^ ^79^, ^80^ ^]^ This involved PCR amplification of specific mtDNA segments: for m.14484T > C, positions 14,260‐14,510. The PCR products for T14484C were treated with *Mva*I restriction enzyme.^[^ ^80^, ^81^ ^]^

To quantify the m.14693A > G mutation, we amplified a 240 bp segment (positions 14608–14847) and digested it with *CviK*I‐1 enzyme, which recognizes a novel site created by the m.14693A > G mutation. Digested and undigested PCR products were then analyzed quantitatively by electrophoresis on 2% agarose gel. The proportions of these products were determined using the Image‐Quant software (Molecular Dynamics, Sunnyvale, CA) after staining with ethidium bromide, enabling us to verify the homoplasmic nature of the m.14693A > G mutation in our subjects.

### Cell Cultures and Culture Conditions

Lymphoblastoid cell lines have been obtained by infecting peripheral blood lymphocytes with Epstein–Barr virus, as described elsewhere.^[^ ^82^ ^]^ The cell lines were utilized from four different family lines, each presenting distinct mtDNA mutations, yet sharing similar mtDNA haplotypes. They also matched in age and sex. This included cell lines from an affected matrilineal relative (HZL017‐III‐3) harboring the tRNA^Glu^ m.14693A > G mutation, another affected matrilineal relative (ZJL855‐III‐3) harboring the ND6 m.14484T > C mutation and a control subject (C101). The cell lines were also included from a patient (ZJL847‐III‐2) with the dual mutations, known for their earlier onset and more severe symptoms of LHON. Their cell lines were cultured in RPMI 1640 medium (Thermo Fisher Scientific, Waltham, MA, USA), enriched with 10% fetal bovine serum (FBS). Additionally, the 143B.TK^–^ cell line was employed, which was maintained in Dulbecco's modified Eagle's medium. This medium was specially formulated with higher glucose (4.5 mg mL^−1^) and pyruvate (0.11 mg mL^−1^) concentrations, and further supplemented with 100 µg mL^−1^ bromodeoxyuridine (BrdU) and 5% FBS. Parallel to this, the *ρ* ^0^206 cell line, a mtDNA‐depleted derivative of 143B.TK^–^, was cultured under similar conditions but with an additional 50 µg mL^−1^ uridine. The transformation of mtDNA‐less *ρ* ^0^206 cells was achieved through cytoplast fusion with the immortalized lymphoblastoid cell lines, as detailed previously.^[^ ^54^, ^83^ ^]^ Subsequently, the cybrids generated from each donor were screened for the presence and concentration of the mutations and their mtDNA copy numbers, following methodologies, as detailed previously.^[^ ^54^, ^83^ ^]^ For biochemical characterization, we selected three cybrids from each donor, ensuring they exhibited homoplasmy for mtDNA mutations and had comparable mtDNA copy numbers. Maintenance of all cybrid cell lines was conducted in the same medium as used for the 143B.TK^–^ cell line.

### Mitochondrial tRNA Analysis

For the detection of pseudouridine residues in tRNA^Glu^, CMCT modification and reverse transcription were performed as detailed previously.^[^ ^59^ ^]^ A DNA primer (5′‐TGGTATTCTCGCACG‐3′) complementary to the 3′ end of the tRNA^Glu^ was 5′ end‐labeled with digoxin (DIG).

For tRNA Northern blot analysis, 10 mg of total mitochondrial RNAs were electrophoresed on an 8% polyacrylamide gel without (native gel) or with (denaturing gel) 8M urea. The gels were then electroblotted onto a positively charged nylon membrane (Roche) for the hybridization analysis with DIG‐labeled oligodeoxynucleotide probes. Oligodeoxynucleotides for tRNA^Glu^, tRNA^Lys^, tRNA^Ser(AGY)^, and tRNA^Leu(UUR)^ have been described elsewhere.^[^ ^84^, ^85^ ^]^ Hybridization and density quantification in each band were carried out as described.^[^ ^23^ ^]^

The S1 nuclease cleavage analysis was performed as detailed elsewhere.^[^ ^86^, ^87^, ^88^ ^]^ In brief, 2 µg of total RNAs were incubated with 1 µg µL^−1^ total yeast tRNA and 1U µL^−1^ S1 nuclease (Thermofisher) in the 5 µL reaction buffer containing 40 mM sodium acetate (pH 4.5), 300 mM NaCl and 2 mM ZnSO_4_. Reaction mixtures were incubated at 28°C for indicated times and quenched by adding 5 µL loading buffer. Samples were electrophoresed through a 10% denaturing polyacrylamide gel with 8 M urea and then electroblotted onto a positively charged nylon membrane for hybridization analysis with DIG‐labeled oligodeoxynucleotide probes as described above.

For Angiogenin Cleavage Assay, 15 µg total RNA extracted from cell lines was used for the cleavage reaction with 2.5 µg mL^−1^ recombinant angiogenin in the buffer containing 30 mM HEPES (pH 7.4), 30 mM NaCl, and 0.01% bovine serum albumin. Mixtures were incubated at 37 °C for the indicated times and quenched by adding 5 µL of gel loading buffer. The products were electrophoresed through a denaturing polyacrylamide gel and stained with methylene blue. Cleavage products of human mitochondrial RNAs were resolved in 15% denaturing polyacrylamide gels with 8 M urea, electroblotted, and hybridized with a DIG‐labeled oligonucleotide probe specific for the tRNA^Pro^ and tRNA^Glu^.^[^ ^89^ ^]^

### UV Melting Assay

UV melting assays were carried out as previously described.^[^ ^59^, ^87^ ^]^ The wild type and mutant tRNA^Glu^ transcripts were generated by in vitro transcription by T7 RNA polymerase (Promega) using synthetic DNA oligonucleotides as templates.

The sequences of oligonucleotides were 5′AGCTAATACGACTCACTATAGGGAGACAAGAACCTGATGAGTCCGTGAGGACGAAACGGTACCCGGTACCGTCGTTCTTGTAGTTGAAATACAACGATGGTTTTTCATATCATTGGTCGTGGTTGTAGTCCGTGCGAGAATACCA‐3′ (wild type); 5′AGCTAATACGACTCACTATAGGGAGACAAGAACCTGATGAGTCCGTGAGGACGAAACGGTACCCGGTACCGTCGTTCTTGTAGTTGAAATACAACGATGGTTTTTCATATCATTGGTCGTGGCTGTAGTCCGTGCGAGAATACCA‐3′ (mutant).

The tRNA^Glu^ transcripts were diluted in the buffer including 50 mM sodium phosphate (pH 7.0), 50 mM NaCl, 5 mM MgCl_2_, and 0.1 mM EDTA. Absorbance versus melting temperature curves was measured at 260 nm with a heating rate of 1 °C/min from 25 to 95 °C through Agilent Cary 100 UV Spectrophotometer.

### Western Blot Analysis

Western blotting analysis was performed as described elsewhere.^[^ ^54^ ^]^ Twenty micrograms of total proteins obtained from various cell lines were electrophoresed through 12% bis–Tris SDS‐polyacrylamide gels. Afterward, the gels were electroblotted onto polyvinylidene difluoride (PVDF) membrane for hybridization. The antibodies used for this investigation were from Abcam [ND1(ab74257), ND3(ab170681), CO1(ab14705) and TOMM20/TOM20(ab56783)], Novus [ND6(NBP2‐94464)], ABclonal [ND2(A6180), ND4L(A17971), CO3(A9939), ATP6(A23150), LC3B(A19665), SQSTM1/p62(A19700), Parkin(A0968) and PINK1(A24745)] and Proteintech [CO2(55070‐1‐AP), Catalase(19792‐1‐AP), SOD2(66474‐1‐Ig), SOD1(67480‐1‐Ig), Bcl‐XL(26967‐1‐AP), Caspase9(66169‐1‐Ig), Caspase3(66470‐2‐Ig), CytC(10993‐1‐AP), BAD(67830‐1‐Ig), BAX9(60267‐1‐Ig), BCL2L13(16612‐1‐AP), GAPDH(60004‐1‐Ig), ATG5(66744‐1‐Ig), ATG7(67341‐1‐Ig), ATG12(11264‐1‐AP), and Beclin‐1(66665‐1‐Ig)]. Peroxidase Affinipure goat anti‐mouse IgG and goat anti‐rabbit IgG (Beyotime) were used as secondary antibodies, and protein signals were detected using the ECL system (CWBIO). Quantification of density in each band was performed as detailed previously.^[^ ^54^ ^]^

### Measurement of ROS Production

The levels of ROS generation by mitochondria in various cell lines were analyzed using the mitochondrial superoxide indicator MitoSOX‐Red following the procedures as detailed previously.^[^ ^47^, ^50^, ^90^ ^]^

### ATP Measurement

The CellTiter‐Glo® luminescent cell viability assay kit (Promega) was used for the measurement of cellular and mitochondrial ATP levels according to the modified instructions of the manufacturer.^[^ ^54^, ^91^ ^]^

### Measurement of Oxygen Consumption

The oxygen consumption rates (OCRs) in cybrid cell lines were measured with a Seahorse Bioscience XF‐96 Extracellular Flux Analyzer (Seahorse Bioscience), as detailed elsewhere.^[^ ^55^, ^92^ ^]^

### Apoptosis Assay

Apoptosis in the cybrids treated with or without gentamycin was determined using the Annexin V‐FITC Apoptosis Kit (Bio Legend, 640914) according to manufacturer's protocol.^[^ ^93^ ^]^ A total of 1 × 10^6^ cells mL^−1^ were seeded in six‐well culture plates and treated with or without gentamycin for 24 h. Then, all of the harvested cells were stained with Annexin V and propidium iodide (PI) for 15 min at room temperature and subsequently analyzed via flow cytometry (FACS Calibur, BD Biosciences). The data were analyzed using FlowJo software, as detailed elsewhere.^[^ ^90^ ^]^

The TUNEL assay was carried out using the One Step TUNEL Apoptosis Assay Kit (C1086, Beyotime, Shanghai, China) according to the manufacturer's protocol. Briefly, cybrid cells were seeded at a density of 2 × 10^4^ cells per well into a V‐bottomed 24‐well microplate overnight. After they were washed twice with PBS, the cells were fixed with freshly prepared 4% paraformaldehyde in PBS for 60 min at RT, permeabilized with 0.1% Triton X‐100 (Sigma‐Aldrich) for 2 min on ice, and incubated in the TUNEL reaction mixture for 60 min at 37 °C. Samples were analyzed under a fluorescence microscope (FV3000, OLYMPUS).

### Assessment of Autophagic Activity

Cybrid cell lines were cultured in Dulbecco's Modified Eagle Medium (DMEM) supplemented with 10% fetal bovine serum. Cells were maintained at 37 °C in a humidified atmosphere containing 5% CO_2_. For autophagy assessment in live‐cell imaging‐compatible culture dishes, cells were transfected with the mCherry‐GFP‐LC3B plasmid using Lipofectamine 3000, according to the manufacturer's instructions. Post‐transfection, cells were incubated for 48 hours to allow for adequate expression of the fluorescent proteins. Cells were examined using a confocal fluorescence microscope (Olympus Fluoview FV1000) with three lasers (Ex/Em = 550/570 and 492/520). In fused mature autolysosomes, only LC3B‐mCherry emits light as the GFP signal quenches in acidic conditions. ImageJ was used to assist in counting and analysis the signals of mature autolysosomes (red).

### Statistical Analysis

Statistical analysis was carried out using the unpaired, two‐tailed Student's t‐test. Differences were considered significant at a p < 0.05.

## Conflict of Interest

The authors declare no conflict of interest.

## Author Contributions

L.J. and D.G. contributed equally as joint first authors. L.J., Y.J., M.G. and M.W. conceived and designed the experiments. L.J., D.G., W.H., L.Z., N.W., S.X., and Y.W. performed the experiments and analyzed the data. L.J. and D.G. prepared the initial draft of the manuscript. M‐X.G. made equal contribution in formal analysis, project administration, writing, review and editing the manuscript. G.E., Y.J., and M.W. made the final version of the manuscript. All authors reviewed the manuscript.

## Supporting information



Supporting Information

## Data Availability

The data that support the findings of this study are available in the supplementary material of this article.
