# Supplementary material for: Mitochondrial tRNAGlu 14693A > G Mutation, an “Enhancer” to the Phenotypic Expression of Leber's Hereditary Optic Neuropathy
Source: Adv Sci (Weinh). 2024 Sep 12;11(41):2401856. doi: 10.1002/advs.202401856 (PMC11538713; doi:10.1002/advs.202401856)
Supplement: Supplementary file 1 — Supporting Information [file ADVS-11-2401856-s001.docx]

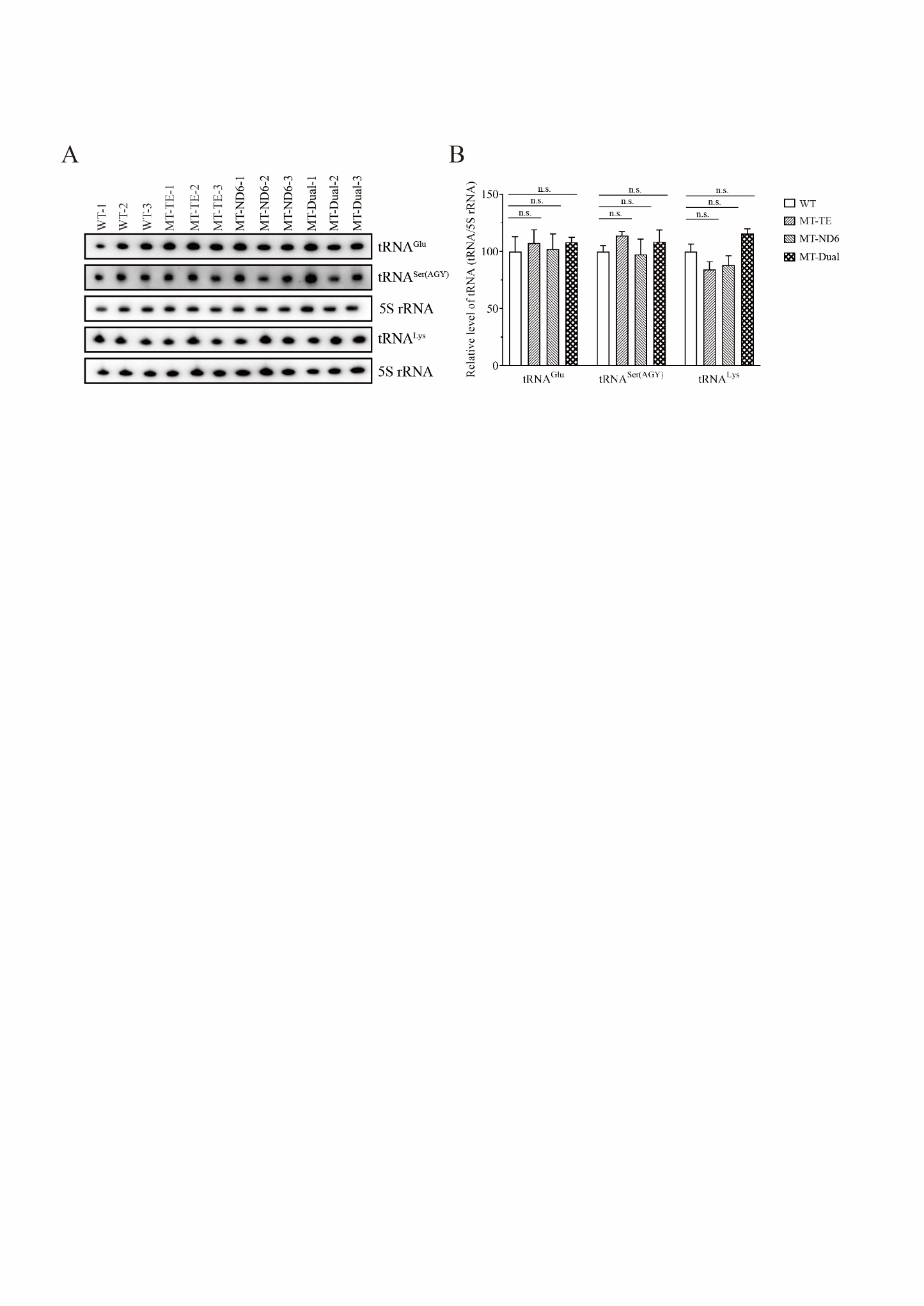


**Figure S1** Northern blot analysis of mitochondrial tRNA under denaturing condition. (A) Ten μg of total mitochondrial RNAs from various cell lines were electrophoresed through a denaturing polyacrylamide gel, electroblotted and hybridized with DIG-labeled oligonucleotide probes specific for the tRNA^Glu^, tRNA^Ser(AGY)^, tRNA^Lys^ and 5S rRNA, respectively. (B) Quantification of tRNA levels. Average relative tRNAs content per cell, was normalized to the average content per cell of reference 5S rRNA in MT-TE, MT-ND6, MT-DUAL and WT cybrids.


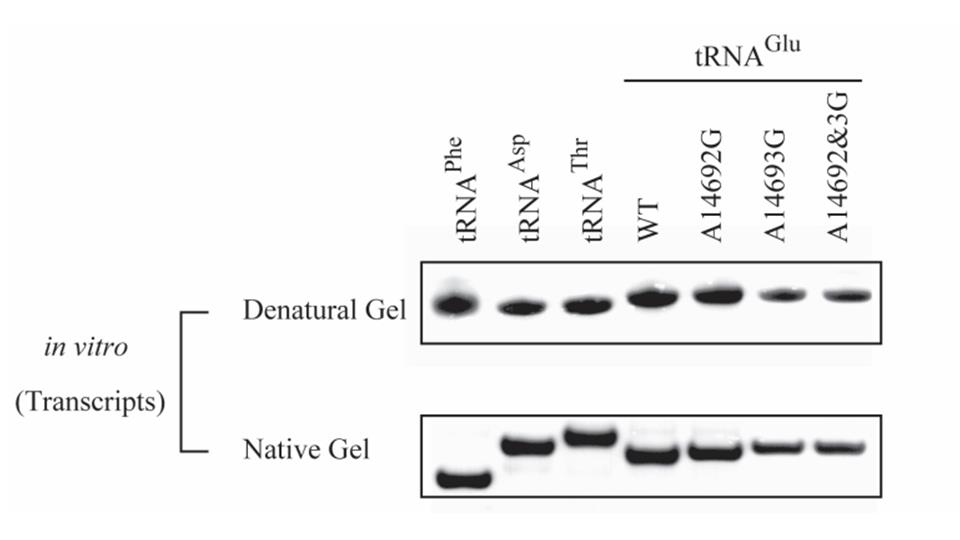


**Figure S2** Analysis of conformation change by Northern blot PAGE analysis under denaturing or native conditions. The transcripts of wild-type and mutant tRNA^Glu^ (72 nt), tRNA^Phe^ (74 nt), tRNA^Asp^ (71 nt), and tRNA^Thr^ (69 nt) were electrophoresed through native or denaturing polyacrylamide gel stained with Methylene Blue.

**Table S1** Summary of clinical data of 3 patients carrying both the m.14693A>G mutation and m.14484T>C mutation.

| Proband | Gender | Age of onset (years) | Vision acuity | | Level of vision impairment | mtDNA haplotype |
| --- | --- | --- | --- | --- | --- | --- |
|  |  |  | OD | OS |  |  |
| ZJL541-IV-1 | M | 5 | 0.04 | CF/10cm | Profound | N9a2 |
| ZJL733-III-9 | M | 7 | 0.04 | 0.04 | Severe | D4e1 |
| ZJL847-III-2 | M | 11 | 0.04 | 0.05 | Severe | Y2 |
